# Does Magnesium Affect Sex Hormones and Cardiometabolic Risk Factors in Patients with PCOS? Findings from a Systematic Review and Meta-Analysis

**DOI:** 10.3390/medicina61020280

**Published:** 2025-02-06

**Authors:** Ahmed Abu-Zaid, Mooza M. Alzayed, Suha Jafar Albahrani, Abdullah Almaqhawi, Mona Ahmed Al Shaikh, Saeed Baradwan, Nawaf Abdulaziz Almudiheem, Mohammed Abuzaid, Heba M. Adly, Saleh A. K. Saleh, Osama Alomar

**Affiliations:** 1 Department of Biochemistry and Molecular Medicine, Alfaisal University, Riyadh 11533, Saudi Arabia; 2 College of Medicine and Medical Sciences, Arabian Gulf University, Manama 329, Bahrain; 3 Department of Family and Community Medicine, College of Medicine, King Faisal University, Al Hofuf 31982, Saudi Arabia; 4 Department of Family Medicine, Eastern Health Cluster, Dammam 32253, Saudi Arabia; 5 Department of Obstetrics and Gynecology, King Faisal Specialist Hospital and Research Center, Jeddah 23433, Saudi Arabia; 6 College of Medicine, Al-Imam Muhammad Ibn Saud Islamic University, Riyadh 13317, Saudi Arabia; 7 Department of Obstetrics and Gynecology, Al Birk General Hospital, Al Birk 63525, Saudi Arabia; 8 Department of Community Medicine and Pilgrims Healthcare, Faculty of Medicine, Umm Al-Qura University, Makkah 24382, Saudi Arabia; 9 Department of Biochemistry, Faculty of Medicine, Umm Al-Qura University, Makkah 24382, Saudi Arabia; 10 Department of Obstetrics and Gynecology, King Faisal Specialist Hospital and Research Center, Riyadh 12713, Saudi Arabia

**Keywords:** magnesium, cardiometabolic risk factors, sex hormones, polycystic ovarian syndrome

## Abstract

*Background and Objectives*: Polycystic ovary syndrome (PCOS) is a common endocrine disorder associated with various cardiometabolic risk factors, including insulin resistance, dyslipidemia, hypertension, and obesity, which contribute to an increased risk of cardiovascular diseases. This inaugural systematic review and meta-analysis of randomized controlled trials (RCTs) evaluated the impact of magnesium supplementation on various cardiometabolic risk factors and hormonal parameters in patients with polycystic ovary syndrome (PCOS). *Materials and Methods*: We systematically searched the MEDLINE, Web of Science, Scopus, and Cochrane databases until 30 March 2024 for studies comparing magnesium supplementation to control in improving cardiometabolic and hormonal factors in PCOS patients. Endpoints were summarized as mean differences (MD) and 95% confidence intervals (CIs) in a random-effects model. *Results*: The primary search yielded 176 studies. After screening, six studies met our inclusion criteria. Our meta-analysis showed no significant effects of magnesium supplementation on cardiometabolic risk factors and hormonal parameters in patients with PCOS. *Conclusions*: Magnesium supplementation does not appear to influence the cardiometabolic and hormonal factors in PCOS patients. Further rigorous RCTs are needed to strengthen the evidence and support comprehensive analysis in this area. PROSPERO database (CRD42024526110).

## 1. Introduction

Polycystic ovary syndrome (PCOS) stands as a prevalent endocrine disorder among women of reproductive age, often cited as the primary reason for infertility stemming from anovulation [1]. According to the World Health Organization, approximately 116 million women globally are affected by PCOS, with its prevalence ranging from 2% to 26% worldwide [2]. PCOS clinically presents with signs such as irregular or absent ovulation, evident hyperandrogenism either clinically or through biochemical means, and/or the presence of polycystic ovaries [3].

Studies have demonstrated that women with PCOS experience a lower quality of life compared to healthy individuals, and even when compared to women with other gynecological conditions [4]. Potential contributors to the diminished quality of life in PCOS include infertility, irregular menstrual cycles, hirsutism, acne, hair loss, as well as anxiety and depression [5].

Furthermore, PCOS is linked with various endocrine and metabolic irregularities. These include conditions such as hyperinsulinemia, hyperglycemia, glucose intolerance, dyslipidemia, and obesity, all of which are recognized components of metabolic syndrome [6]. These correlations contribute to the pathophysiology of the PCOS by exacerbating insulin resistance and increasing cardiovascular risk [7]. While the exact cause of this syndrome remains incompletely understood, PCOS is frequently accompanied by a condition of chronic low-grade inflammation and oxidative stress (OS), which is strongly linked to additional clinical and metabolic disorders [8,9]. Several studies have shown that exposure to OS reduces insulin-stimulated glucose uptake, glycogen synthesis, and protein synthesis [10,11]. However, the exact mechanism underlying this effect remains incompletely understood [12].

Both genetic predispositions and environmental influences, including dietary habits, play significant roles as contributing factors to PCOS [13]. Lifestyle modifications stand as the initial treatment approach for PCOS, and minor adjustments in lifestyle habits such as diet, exercise, and behavior have been shown to enhance metabolic function, ovulation, fertility, and mood [14,15,16]. In addition to lifestyle modifications, pharmacological therapies such as metformin and anti-androgens are proven strategies for reducing cardiometabolic risk [17]. Dietary approaches like the DASH diet and the Mediterranean diet, along with regular physical activity, are effective in improving metabolic health and mitigating the cardiometabolic complications associated with PCOS [18,19]. Furthermore, there has been significant interest in the use of nutritional supplements for managing PCOS [20,21,22]. Among these, minerals such as magnesium have shown promise in the prevention and management of PCOS. Magnesium supplementation can improve insulin sensitivity, reduce inflammation, and support overall metabolic health, making it a valuable addition to lifestyle interventions aimed at alleviating PCOS symptoms [23].

Magnesium, the second most abundant intracellular cation in the human body, plays a crucial role in both health and disease [24]. It has been revealed that magnesium functions as a vital antioxidant, serving as a cofactor for numerous enzymes involved in cell membrane stabilization and the reduction in oxidative stress [25]. Previous studies indicate that women with PCOS tend to consume fewer magnesium-rich foods [26,27], and they have lower serum magnesium levels than healthy people [28]. Magnesium plays a role in reducing menstrual pain and cramps and is crucial for protein formation, cell growth, and division [29]. Additionally, magnesium may improve skin lesions and acne by enhancing collagen production, while low magnesium intake can lead to inflammation [30].

Some studies demonstrated beneficial effects of consuming magnesium on improving insulin sensitivity in people with hyperglycemia [31], type 2 diabetes (T2D) [32], metabolic syndrome [33] and PCOS [34]. Shahmoradi et al. also showed that magnesium supplementation can not only reduce serum insulin level and insulin resistance, but it can also decrease total cholesterol, low-density lipoprotein (LDL), and fasting blood sugar (FBS) in women with PCOS compared to the placebo group [34]. While other studies have shown no significant effects on glycemic variables and lipid profiles following magnesium supplementation in PCOS patients [35,36].

To the best of our knowledge, there is no systematic review and meta-analysis study to summarize and clarify the net effect of magnesium supplementation on cardiometabolic risk factors. Thus, in our current first-ever systematic review and meta-analysis, we summarized the effects of magnesium supplementation on cardiometabolic risk factors in randomized clinical trials.

## 2. Methods

### 2.1. Study Protocol

The findings of this comprehensive systematic review and meta-analysis were meticulously reported, adhering to the Preferred Reporting Items for Systematic reviews and Meta-Analyses (PRISMA) guidelines [37] and registered in the International Prospective Register of Systematic Reviews (PROSPERO) database (CRD42024526110). The study protocol followed the PRISMA 2020 guideline and Cochrane Handbook recommendations, ensuring transparency and reliability in the research process [38].

### 2.2. Search Strategy

A systematic search of major web-based databases, including MEDLINE, Web of Science, Scopus, and Cochrane, was conducted up to 30 March 2024, using specific search terms related to magnesium and PCOS. This search aimed to identify relevant randomized controlled trials (RCTs) assessing the impact of magnesium supplementation on PCOS patients. Furthermore, the reference lists of included studies and previous reviews were manually examined to uncover additional eligible research, ensuring comprehensive coverage and minimizing publication bias. The search strategy was meticulously crafted with input from a skilled librarian, experts in knowledge synthesis research methods, and the research team, employing tailored combinations of subject terms and keywords for each database. The search terms were composed of: “Polycystic Ovary Syndrome” OR “PCOS” AND “Magnesium” OR “Magnesium sulfate” OR “Magnesium supplementation”. The complete search strategy for each database and search syntaxes are presented in Supplementary File S1.

### 2.3. Inclusion and Exclusion Criteria

The inclusion criteria for this analysis were guided by the PICOS framework. The population consisted of individuals diagnosed with polycystic ovary syndrome (PCOS) according to the Rotterdam criteria, which require the presence of two out of the following three features: (1) clinical or biochemical hyperandrogenism, (2) ovulatory dysfunction, and (3) polycystic ovaries on ultrasound [39]. The interventions focused on magnesium supplementation, irrespective of the specific type, duration, or dosage. Comparisons were made against either placebo or no intervention groups.

The outcomes assessed included various cardiometabolic risk factors. Anthropometric measurements included body weight (BW), waist circumference (WC), and body mass index (BMI). Glycemic indices were evaluated through fasting blood glucose (FBS), insulin levels, and the homeostasis model assessment of insulin resistance (HOMA-IR). Lipid profiles encompassed total cholesterol (TC), triglycerides (TG), low-density lipoprotein (LDL), and high-density lipoprotein (HDL). Blood pressure measurements included systolic blood pressure (SBP) and diastolic blood pressure (DBP). Lastly, sex-related hormones assessed included dehydroepiandrosterone (DHEA), testosterone, the free androgen index (FAI), and sex hormone-binding globulin (SHBG).

Only randomized controlled trials (RCTs) were considered for inclusion, with no restrictions on language, sample size, age, ethnicity, or publication date. Both published and unpublished studies were reviewed, while exclusion criteria included non-randomized studies, such as case–control, case series, cohort, and cross-sectional studies.

### 2.4. Study Selection and Data Extraction

Two investigators (M. A. and O. A.) meticulously screened the titles and abstracts of potential studies to identify those meeting the eligibility criteria. Upon obtaining full-text copies of relevant articles, they independently extracted key information such as author details, publication year, study location, participant demographics including age and BMI, sample size, and specifics regarding magnesium supplementation. Any discrepancies were resolved through consultation with a third reviewer (A. A-Z.), ensuring accuracy in data collection. Standardized protocols were employed, with interrater agreement established beforehand to ensure consistency in screening and assessment processes. Abstracts and full-texts were reviewed by separate teams, with disagreements resolved promptly by the primary investigator. Additionally, missing data from published articles were diligently pursued through direct correspondence with the respective authors, ensuring comprehensive data inclusion for analysis.

### 2.5. Study Risk of Bias Assessment

The evaluation of trial bias was conducted utilizing the Cochrane risk of bias (ROB) tool, version 2, encompassing five key domains: randomization process, adherence to interventions, handling of missing data, outcome measurement, and selection of reported outcomes [40]. Each domain was meticulously scrutinized, and trials were categorized based on the level of bias risk identified. Trials were classified as having “low risk” if all domains exhibited minimal bias, “some concerns” if at least one domain raised issues without reaching a high bias level, or “high risk” if one or more domains indicated significant bias or if concerns spanned multiple domains. This evaluation process was carried out independently by two assessors (S. B. and M. M. A.), with any discrepancies resolved through consultation and consensus-building among the team.

### 2.6. Statistical Analysis

The meta-analysis employed STATA version 17.0 software (StataCorp, College Station, TX, USA), utilizing a random-effects model to analyze continuous outcomes, expressed as mean differences with 95% confidence intervals (CIs). Statistical significance was determined by *p*-values < 0.05. Heterogeneity among studies was evaluated via the I^2^ statistic > 50% [41]. Quantitative data underwent analysis for changes from baseline to follow-up, with missing standard deviations estimated based on available confidence intervals or other measures. Forest plots were utilized to present pooled effect sizes, with a significance threshold set at *p* < 0.05 for effect size estimation.

## 3. Results

### 3.1. Summary of Literature Search, Study Selection, and Risk of Bias Assessment

**Figure 1:**
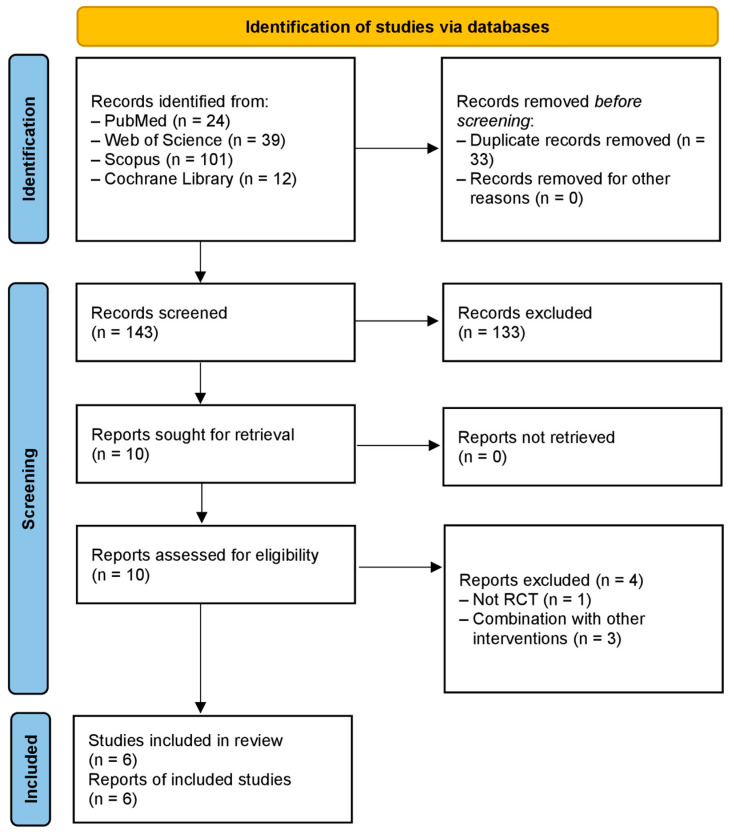
The PRISMA flow diagram for literature search and study selection.

**Figure 2:**
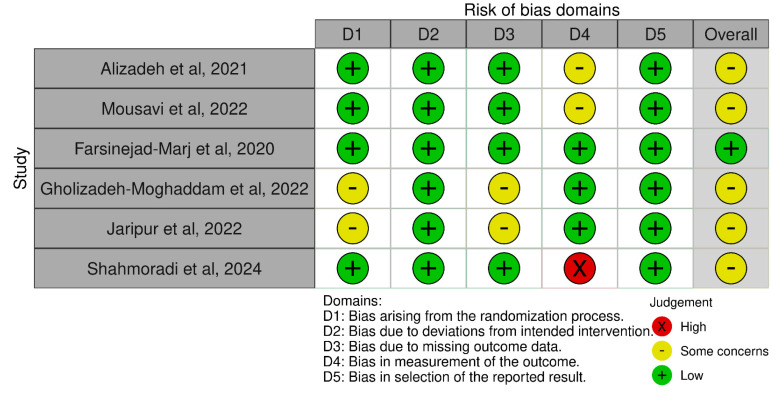
Risk of bias assessment of the included studies.

After conducting a thorough literature search, we initially found 176 publications relevant to our study. Through the screening process, which involved removing duplicates and non-relevant abstracts, we narrowed down our selection to 10 full-text articles suitable for inclusion in our systematic review and meta-analysis. Among these, some were excluded due to inappropriate interventions or non-RCT designs, resulting in a final inclusion of 6 studies [34,35,42,43,44,45]. Our systematic approach, as depicted in Figure 1, adhered to the PRISMA guidelines, ensuring transparency and rigor in our research selection process. Figure 2 summarizes the quality assessment of the studies included. Most studies exhibited “some concerns of bias” due to potential issues related to randomization processes, missing outcome data, or the measurement of outcomes.

### 3.2. Characteristics of Included Studies and Risk of Bias Assessment

We included six articles from four RCTs (Alizadeh et al., 2021 [42] and Mousavi et al., 2022 [45], as well as Gholizadeh-Moghaddam et al., 2022 [43] and Jaripur et al., 2022 [44], reported different variables from the same trials). These trials were published between 2020 and 2024. A total of 248 PCOS patients participated in the meta-analysis, with ages ranging from 25 to 32 years and BMIs varying between 26 and 30 kg/m^2^. All studies were conducted in Iran. The duration of the supplementation period ranged from 8 to 20 weeks, with all studies administering 250 mg/d of magnesium for supplementation. Table 1 displays the main characteristics of each study included in the meta-analysis.

### 3.3. Effect of Magnesium on Cardiometabolic (Anthropometric, Glycemic, Lipid, and Blood Pressure) Risk Factors

**Figure 3:**
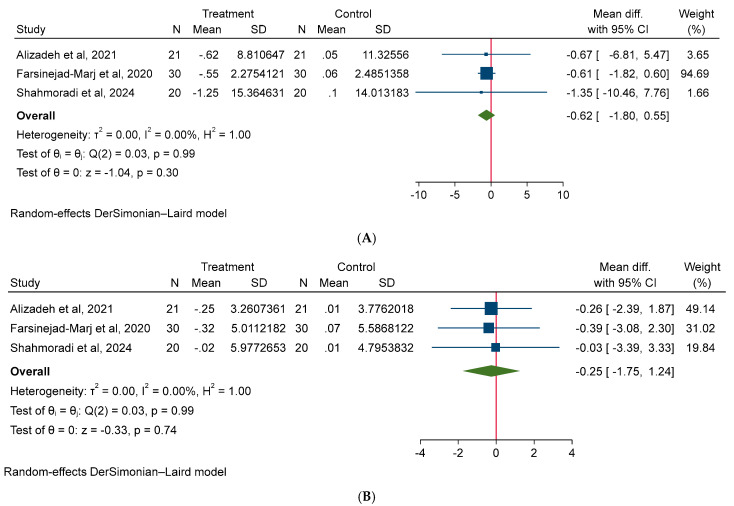

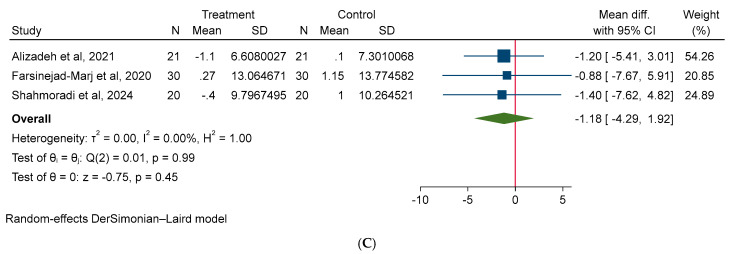
The effect of magnesium supplementation on anthropometric indices: (**A**) body weight, (**B**) body mass index, and (**C**) waist circumference in patients with PCOS.

**Figure 4:**
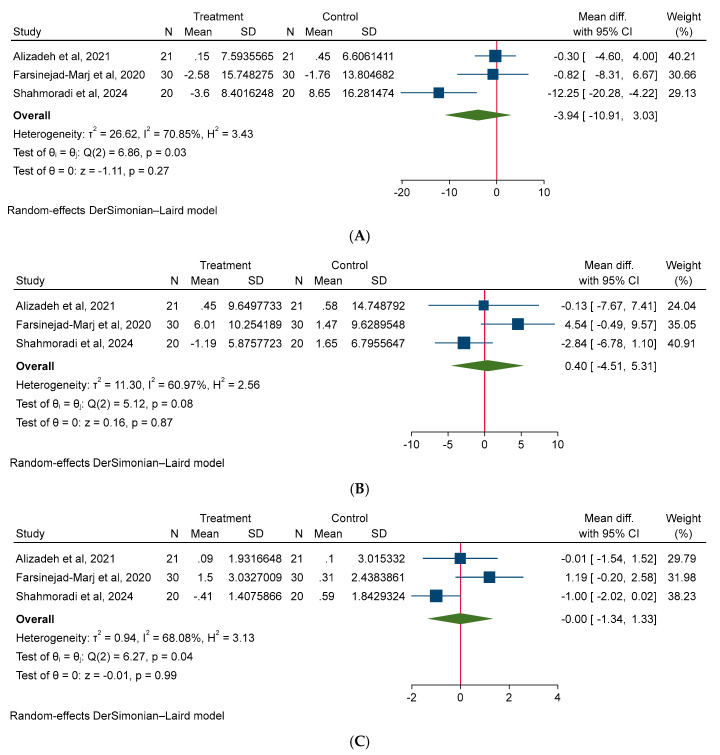
The effect of magnesium supplementation on glycemic parameters: (**A**) FBG, (**B**) insulin, and (**C**) HOMA-IR in patients with PCOS.

**Figure 5:**
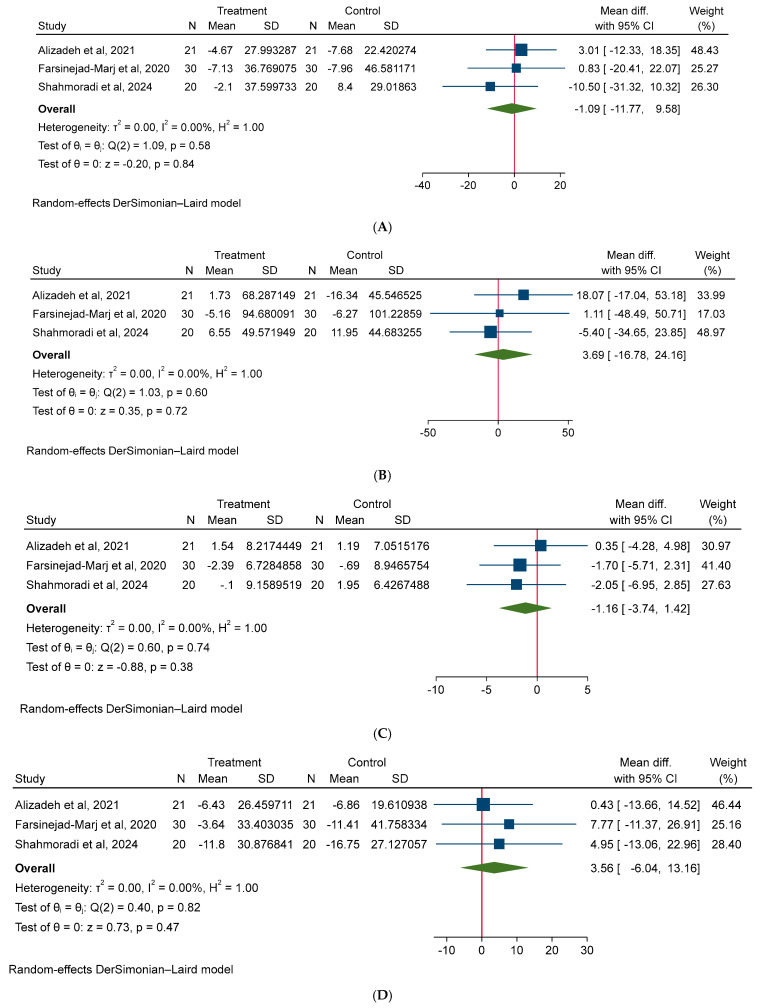
The effect of magnesium supplementation on lipid profile: (**A**) TC, (**B**) TG, (**C**) HDL, and (**D**) LDL in patients with PCOS.

**Figure 6:**
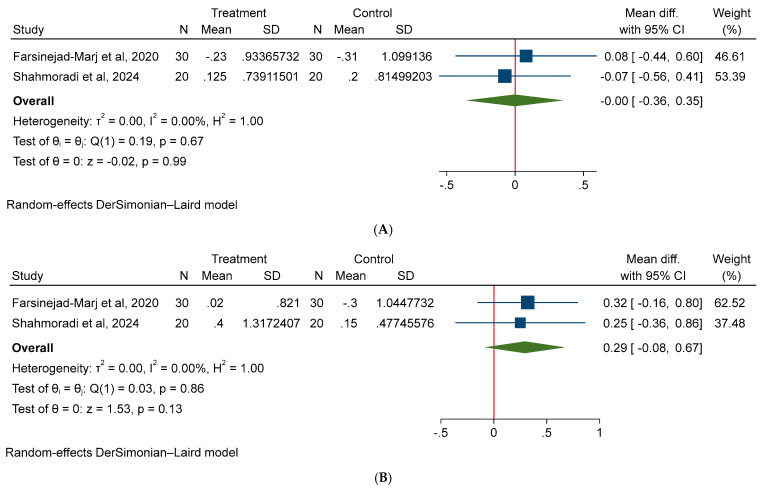
The effect of magnesium supplementation on blood pressure: (**A**) SBP and (**B**) DBP in patients with PCOS.

A random-effect model was used for all meta-analyses. The results of the meta-analysis shown non-significant effect of magnesium on body weight (MD: −0.62 kg; 95% CI: −1.80, 0.55; I^2^ = 0.00%), BMI (MD: −0.25 kg/m^2^; 95% CI: −1.75, 1.24; I^2^ = 0.00%), and WC (MD: −1.18 cm; 95% CI: −4.29, 1.92; I^2^ = 0.00%) in PCOS patients (Figure 3). Additionally, our meta-analysis model did not detect a significant effect of magnesium intake on glycemic parameters, such as FBS (MD: −3.94 mg/dL; 95% CI: −10.91, 3.03; I^2^ = 70.85%), insulin (MD: 4.0 µU/mL; 95% CI: −4.51, 5.31; I^2^ = 60.97%), HOMA-IR (MD: −0.00; 95% CI: −1.34, 1.33, I^2^ = 68.08%) (Figure 4). Moreover, no significant effect of magnesium intake was noted on lipid parameters, such as TC (MD: −1.09 mg/dL; 95% CI: −11.77, 9.58; I^2^ = 0.00%), TG (MD: 3.69 mg/dL; 95% CI: −16.78, 24.16; I^2^ = 0.00%), HDL (MD: −1.16 mg/dL; 95% CI: −3.74, 1.42; I^2^ = 0.00%), LDL (MD: 3.56 mg/dL; 95% CI: −6.04, 13.16, I^2^ = 0.00%) (Figure 5). Lastly, no significant effect of magnesium intake was exhibited on BP parameters, such as SBP (MD: −0.00 mmHg; 95% CI: −0.36, 0.35; I^2^ = 0.00%), and DBP (MD: 0.29 mmHg; 95% CI: −0.07, 0.67; I^2^ = 0.00%) (Figure 6).

### 3.4. Effect of Magnesium on Hormonal Factors in PCOS Patients

**Figure 7:**
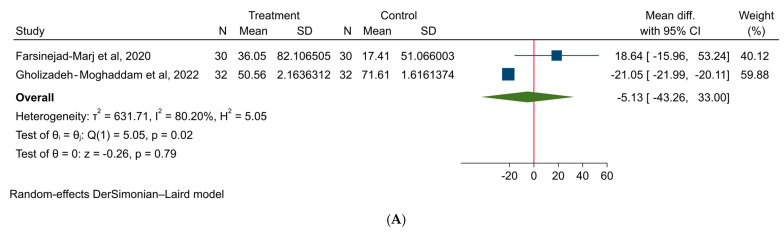

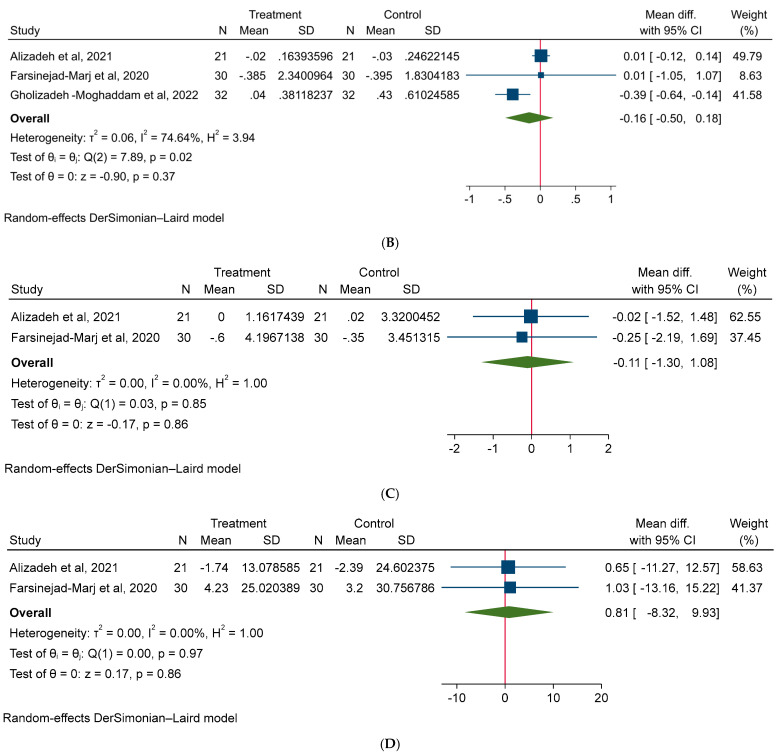
The effect of magnesium supplementation on hormonal factors: (**A**) DHEA, (**B**) testosterone, (**C**) FAI, and (**D**) SHBG in patients with PCOS.

Our meta-analysis indicated that magnesium supplementation does not significantly affect DHEA (MD: −5.13 µg/dL; 95% CI: −43.26, 33.00, I^2^ = 80.20%), testosterone (MD: −0.16 ng/dL; 95% CI: −0.50, 0.18, I^2^ = 76.64%), FAI (MD: −0.11; 95% CI: −1.30, 1.08, I^2^ = 0.00%), and SHBG (MD: 0.81 nmol/L; 95% CI: −8.32, 9.93, I^2^ = 0.00%) levels in patients with PCOS (Figure 7).

### 3.5. Effect of Magnesium on Quality of Life of PCOS Patients

There was only one study examining the impact of magnesium on the quality of life among PCOS patients, preventing us from conducting a meta-analysis in this area. Nevertheless, the findings from these studies suggested that magnesium supplementation could enhance physical functioning, alleviate limitations stemming from physical or emotional issues, boost energy levels, promote emotional well-being, improve social interactions, enhance overall health, and contribute to a better total quality of life.

## 4. Discussion

The present systematic review and meta-analysis was designed to evaluate the effects of magnesium supplementation on cardiometabolic risk factors and sex hormones levels in patients with PCOS. All included trials in our study were evaluated based on the Rotterdam diagnostic criteria for PCOS. The participants in all included studies did not show statistical differences in age or BMI. Results showed no significant effects of magnesium supplementation on lipid profile, blood pressure, anthropometric and glycemic indices. Also, similar results were revealed regarding sex hormones levels. This systematic review and meta-analysis highlights that there is insufficient evidence to make recommendations on magnesium supplementation in PCOS women. Therefore, to answer the research question posed, more rigorous studies are required; well-designed and high quality randomized clinical trials with bigger sample sizes and longer duration.

There is increasing evidence suggesting that defects in insulin actions or in the insulin signaling pathways play a central role in the development of PCOS. In fact, a majority of women with PCOS experience metabolic insulin resistance, which is partly attributable to genetic predisposition and partly to secondary factors such as obesity [46]. However, Shirazi et al. reported that this resistance to insulin is independent of fat [47]. Insulin is a vital hormone that regulates the cellular metabolism in many tissues in the human body. Insulin resistance is defined as a reduction in tissue response to insulin stimulation hence insulin resistance (IR) is characterized by defects in uptake and oxidation of glucose, a decrease in glycogen synthesis, and, to a smaller degree, the ability to suppress lipid oxidation [48]. IR results in elevated free androgen levels, leading to alterations in follicular development and dysfunction of granulosa cells [49,50]. Elevated insulin levels in PCOS patients decrease serum levels of sex hormone-binding globulin (SHBG), consequently increasing the bioavailability of free testosterone. Additionally, these women often exhibit abnormal gonadotropin concentrations and heightened androgen biosynthesis from both the adrenal glands and ovaries, driven by elevated insulin levels [51]. Moreover, there is a possibility of a significant impact of hyperandrogenism on insulin resistance in PCOS patients [52]. IR can additionally disrupt systemic lipid metabolism, resulting in the dyslipidemia development and the well-recognized lipid triad: (1) elevated plasma TG levels, (2) decreased HDL levels, and (3) the presence of small dense LDLs [48]. Insulin resistance also decreases lipoprotein lipase activity, a major mediator of VLDL clearance [48], and it has been strongly correlated with insulin resistance. In addition, insulin resistance and obesity can induce dyslipidemia [53,54]. Therefore, paying attention to the anthropometric indices as a risk factor for IR and dyslipidemia in PCOS patients is crucial.

From another point of view, PCOS is widely recognized as a pro-inflammatory condition [55]. Physiological hyperglycemia generates increased levels of reactive oxygen species (ROS) from mononuclear cells, which then activate the release of tumor necrosis factor alpha (TNF-α) and increase inflammatory transcription factor NF-kappa B. As a result, the concentrations of TNF-α, a known mediator of insulin resistance, are further increased. The resulting OS creates an inflammatory environment that promotes insulin resistance and contributes to hyperandrogenism [56]. In confirmation of the previous statement, studies suggest that chronic low-grade inflammation also contribute to IR, type 2 diabetes mellitus (T2DM), and cardiovascular disease [57,58]. Hence, evaluating inflammatory markers in conjunction with assessing IR and measuring blood lipids can be notably beneficial.

According to the previous studies, women with PCOS tend to exhibit elevated serum levels of copper, cobalt, chromium, and iron, alongside lower concentrations of selenium and magnesium [59]. Magnesium serves as the primary intracellular divalent cation and plays a crucial physiological role in numerous functions. in addition, magnesium is essential for the synthesis of nucleic acids and proteins, and is an important cofactor for a large number of enzymes and transporters [60], as a result, it has important effects on energy metabolism and the cardiovascular system [61]. There is evidence suggesting that dietary magnesium deficiencies may increase inflammatory responses. For instance, CRP and pro-inflammatory cytokines have been found to be associated with magnesium status. Conversely, there is substantial evidence supporting the beneficial effects of magnesium supplementation on biomarkers of inflammation and oxidative stress as well [62], while it was co-supplemented with zinc. A meta-analysis of 18 RCTs showed no significant effect of magnesium supplementation on inflammatory markers in adults [63]. Therefore, while inflammatory responses play a crucial role in the pathogenesis and development of IR, the effects of magnesium supplementation on inflammatory markers are controversial, which were not evaluated in the current study.

Sharifi et al. revealed that although magnesium deficiency increased the risk of PCOS, they found no relationship between blood magnesium levels and insulin resistance, insulin levels, fasting blood sugar, and fat disorders [64]. This underscores the complexity of interrelationship between IR, inflammation, oxidative stress, obesity and lipid profile in patients with PCOS, interventions with the goal of evaluating the effects of supplements must consider all these factors in order to increase the precision and reliability of results. On the other hand, previous studies demonstrated that magnesium might have beneficial effects on hyperandrogenism and hirsutism, but participants have used magnesium in combination with other nutrients like vitamin E [65]. 

Overall, the effect of magnesium on cardiometabolic risk factors and sex hormones in PCOS women can be influenced by many factors such as population type or genetic, inclusion and exclusion criteria, baseline characteristics of women, dose and duration of supplementation. According to our knowledge, it was the first systematic review and meta-analysis aimed to investigate the effects of magnesium supplementation alone on cardiovascular risk factors and sex hormones in PCOS patients compared to the control group. This systematic review has some limitations as well. The first is the small number of included studies which did not allow us to implement a subgroup analysis to draw a more resolute conclusion in each of the individual variables. For example, BMI may make a difference in the results and a subgroup analysis based on BMI may help us better clarify the effect of magnesium. Secondly, all the included trials were performed in Iran that weaken generalizability. Thirdly, due to the limited number of included studies, we were unable to perform deeper analyses, such as subgroup analysis. Additionally, because the primary studies evaluated a limited set of variables, we could not provide a comprehensive analysis of the effect of magnesium on oxidative stress and inflammatory factors. Eventually more randomized clinical are needed not only assess the effects of magnesium on cardiometabolic risk factors and sex hormone levels, but also on inflammatory and oxidative stress biomarkers. Additionally, focusing on the underlying mechanisms may provide more appropriate and accurate insights.

## 5. Conclusions

Magnesium supplementation does not appear to influence the cardiometabolic and hormonal factors in PCOS patients. Further rigorous RCTs are needed to strengthen the evidence and support comprehensive analysis in this area.

## Figures and Tables

**Table 1:** The main characteristics of included studies.

Study	Country	n	Magnesium Dose (mg/d)	Magnesium Duration (wk)	Age (Years)		BMI (kg/m^2^)		Main Outcomes *
					Magnesium	Control	Magnesium	Control	
Alizadeh et al., 2021 [42] Mousavi et al., 2022 * [45]	Iran	84	250	8	25.57 ± 4.88	26.2 ± 5.72	27.99 ± 3.22	26.94 ± 3.83	↓Testosterone, ↓WC, ↔BMI, ↔FBS, ↔Insulin, ↔HOMA-IR, ↔TC, ↔TG, ↔HDL, ↔LDL, ↔SHBG, ↔Hirsutism, ↔MDA, ↔TAC, ↔TNF-α, ↔hs-CRP
Farsinejad-Marj et al., 2020 [35]	Iran	60	250	8	26.32 ± 3.92	26 ± 5.06	27.8 ± 5.07	27.72 ± 5.40	↓BMI, ↔Weight, ↔WC, ↔SBP, ↔DBP, ↔FBS, ↔Insulin, ↔QUICKI, ↔HOMA-IR, ↔HOMA-B, ↔TC, ↔TG, ↔HDL, ↔LDL, ↔FSH, ↑LH, ↔FAI, ↓Testosterone, ↔SHBG, ↑DHEA
Gholizadeh-Moghaddam et al., 2022 [43] Jaripur et al., 2022 * [44]	Iran	64	250	10	31.69 ± 5.41	32.44 ± 6.42	26.89 ± 4.68	26.63 ± 4.06	↔Life quality, ↔Hirsutism, ↔Sleep Quality, ↔Testosterone, ↔DHEA
Shahmoradi et al., 2024 [34]	Iran	40	250	20	27.45 ± 5.04	29.00 ± 4.24	29.73 ± 6.44	30.61 ± 5.03	↔BMI, ↔Weight, ↔WC, ↔HC, ↔SBP, ↔DBP, ↓FBS, ↓Insulin, ↓HOMA-IR, ↓TC, ↔TG, ↑HDL, ↓LDL

↓ this symbol is a sign of decreasing variables in the intervention group, ↑ this symbol is a sign of increasing variables in the intervention group, ↔ this symbol indicates that there is no difference between the two groups. FAI: free androgen index; DBP: diastolic blood pressure; DHEA: dehydroepiandrosterone; FBS: fasting blood sugar; HDL: high-density lipoproteins; HC: hip circumference; HOMA-IR: homeostatic model assessment-insulin resistance; hs-CRP: high-sensitivity C-reactive protein; LDL: low-density lipoproteins; TC: total cholesterol; SBP: systolic blood pressure; SHBG: sex hormone-binding globulin; TG: triglycerides; WC: waist circumference; BMI: body mass index. * Alizadeh et al., 2021 [42] and Mousavi et al., 2022 [45], as well as Gholizadeh-Moghaddam et al., 2022 [43] and Jaripur et al., 2022 [44], reported different variables from the same trials.

## Data Availability

All data are available within this manuscript and its Supplementary Materials.

## Supplementary Materials

The following supporting information can be downloaded at: https://www.mdpi.com/article/10.3390/medicina61020280/s1, Supplementary File S1. The exact literature search strategy for databases.
