# Supplementary material for: Does Magnesium Affect Sex Hormones and Cardiometabolic Risk Factors in Patients with PCOS? Findings from a Systematic Review and Meta-Analysis
Source: Medicina (Kaunas). 2025 Feb 6;61(2):280. doi: 10.3390/medicina61020280 (PMC11857371; doi:10.3390/medicina61020280)
Supplement: Supplementary file 1 [file medicina-61-00280-s001.zip › Supplementary File 1_Mg and PCOS_11-02-24.pdf]

Supplementary File 1. The exact literature search strategy for databases.

PICOS EVIDENCE-BASED QUESTION

Groups	Descriptors
Outcome	Polycystic Ovary Syndrome OR Ovary Syndrome, Polycystic OR Syndrome, Polycystic Ovary OR Polycystic ovary disease OR Stein-Leventhal Syndrome OR Stein Leventhal Syndrome OR Syndrome, Stein-Leventhal OR Sclerocystic Ovarian Degeneration OR Ovarian Degeneration, Sclerocystic OR Sclerocystic Ovary Syndrome OR Polycystic Ovarian Syndrome OR Ovarian Syndrome, Polycystic OR Polycystic Ovary Syndrome 1 OR Sclerocystic Ovaries OR Ovary, Sclerocystic OR Sclerocystic Ovary OR hyperandrogenism OR Hypertrichosis OR Hirsutism OR “PCOS” OR “PCO”
Exposure	Magnesium OR “Magnesium sulfate” OR “Magnesium supplementation”
Setting	Randomized controlled trial OR controlled clinical trial OR randomized controlled trials OR random allocation OR double blind method OR single blind method OR clinical trial OR clinical trials OR placebos OR placebo OR random

PUBMED

Number of localized studies: 24
Limits: -
Number of studies after applying limits: 24

	Descriptors	Number of studies reached
**#1**	((((((((((((((((((("polycystic ovary syndrome"[MeSH Terms] OR ("polycystic"[All Fields] AND "ovary"[All Fields] AND "syndrome"[All Fields]) OR "polycystic ovary syndrome"[All Fields]) OR ("polycystic ovary syndrome"[MeSH Terms] OR ("polycystic"[All Fields] AND "ovary"[All Fields] AND "syndrome"[All Fields]) OR "polycystic ovary syndrome"[All Fields] OR ("ovary"[All Fields] AND "syndrome"[All Fields] AND "polycystic"[All Fields]))) OR ("polycystic ovary syndrome"[MeSH Terms] OR ("polycystic"[All Fields] AND "ovary"[All Fields] AND "syndrome"[All Fields]) OR "polycystic ovary syndrome"[All Fields] OR ("polycystic"[All Fields] AND "ovary"[All Fields] AND "disease"[All Fields]) OR "polycystic ovary disease"[All Fields])) OR ("polycystic ovary syndrome"[MeSH Terms] OR ("polycystic"[All Fields] AND "ovary"[All Fields] AND "syndrome"[All Fields]) OR "polycystic ovary syndrome"[All Fields] OR ("stein"[All Fields] AND "leventhal"[All Fields] AND "syndrome"[All Fields]) OR "stein leventhal syndrome"[All Fields])) OR ("polycystic ovary syndrome"[MeSH Terms] OR ("polycystic"[All Fields] AND "ovary"[All Fields] AND "syndrome"[All Fields]) OR "polycystic ovary syndrome"[All Fields] OR ("stein"[All Fields] AND "leventhal"[All Fields] AND "syndrome"[All Fields]) OR "stein leventhal syndrome"[All Fields])) OR ("polycystic ovary syndrome"[MeSH Terms] OR ("polycystic"[All Fields] AND "ovary"[All Fields] AND "syndrome"[All Fields]) OR "polycystic ovary	40194

	syndrome"[All Fields] OR ("syndrome"[All Fields] AND "stein"[All Fields] AND "leventhal"[All Fields]))) OR ("polycystic ovary syndrome"[MeSH Terms] OR ("polycystic"[All Fields] AND "ovary"[All Fields] AND "syndrome"[All Fields]) OR "polycystic ovary syndrome"[All Fields] OR ("sclerocystic"[All Fields] AND "ovarian"[All Fields] AND "degeneration"[All Fields]) OR "sclerocystic ovarian degeneration"[All Fields])) OR ("polycystic ovary syndrome"[MeSH Terms] OR ("polycystic"[All Fields] AND "ovary"[All Fields] AND "syndrome"[All Fields]) OR "polycystic ovary syndrome"[All Fields] OR ("ovarian"[All Fields] AND "degeneration"[All Fields] AND "sclerocystic"[All Fields]))) OR ("polycystic ovary syndrome"[MeSH Terms] OR ("polycystic"[All Fields] AND "ovary"[All Fields] AND "syndrome"[All Fields]) OR "polycystic ovary syndrome"[All Fields] OR ("sclerocystic"[All Fields] AND "ovary"[All Fields] AND "syndrome"[All Fields]) OR "sclerocystic ovary syndrome"[All Fields])) OR ("polycystic ovary syndrome"[MeSH Terms] OR ("polycystic"[All Fields] AND "ovary"[All Fields] AND "syndrome"[All Fields]) OR "polycystic ovary syndrome"[All Fields] OR ("polycystic"[All Fields] AND "ovarian"[All Fields] AND "syndrome"[All Fields]) OR "polycystic ovarian syndrome"[All Fields])) OR ("polycystic ovary syndrome"[MeSH Terms] OR ("polycystic"[All Fields] AND "ovary"[All Fields] AND "syndrome"[All Fields]) OR "polycystic ovary syndrome"[All Fields] OR ("ovarian"[All Fields] AND "syndrome"[All Fields] AND "polycystic"[All Fields]))) OR ("polycystic ovary syndrome"[MeSH Terms] OR ("polycystic"[All Fields] AND "ovary"[All Fields] AND "syndrome"[All Fields]) OR "polycystic ovary syndrome"[All Fields] OR "polycystic ovary syndrome 1"[All Fields])) OR ("polycystic ovary syndrome"[MeSH Terms] OR ("polycystic"[All Fields] AND "ovary"[All Fields] AND "syndrome"[All Fields]) OR "polycystic ovary syndrome"[All Fields] OR ("sclerocystic"[All Fields] AND "ovaries"[All Fields]) OR "sclerocystic ovaries"[All Fields])) OR ("polycystic ovary syndrome"[MeSH Terms] OR ("polycystic"[All Fields] AND "ovary"[All Fields] AND "syndrome"[All Fields]) OR "polycystic ovary syndrome"[All Fields] OR ("ovary"[All Fields] AND "sclerocystic"[All Fields]))) OR ("polycystic ovary syndrome"[MeSH Terms] OR ("polycystic"[All Fields] AND "ovary"[All Fields] AND "syndrome"[All Fields]) OR "polycystic ovary syndrome"[All Fields] OR ("sclerocystic"[All Fields] AND "ovary"[All Fields]) OR "sclerocystic ovary"[All Fields])) OR ("hyperandrogenism"[MeSH Terms] OR "hyperandrogenism"[All Fields])) OR ("hypertrichosis"[MeSH Terms] OR "hypertrichosis"[All Fields])) OR ("hirsutism"[MeSH Terms] OR "hirsutism"[All Fields])) OR PCOS[All Fields]) OR PCO[All Fields]	
**#2**	"magnesium"[MeSH Terms] OR "magnesium"[All Fields] OR "magnesium s"[All Fields] OR "magnesiums"[All Fields] OR ("magnesium sulphate"[All Fields] OR "magnesium sulfate"[MeSH Terms] OR ("magnesium"[All Fields] AND "sulfate"[All Fields]) OR "magnesium sulfate"[All Fields]) OR (("magnesium"[MeSH Terms] OR "magnesium"[All Fields] OR "magnesium s"[All Fields] OR "magnesiums"[All Fields]) AND ("supplemental"[All Fields] OR "supplementating"[All Fields] OR "supplementation"[All Fields] OR "supplementation s"[All Fields] OR "supplementations"[All Fields] OR "supplemention"[All Fields]))	121703
**#3**	(((((((("Randomized Controlled Trial"[Publication Type] OR "Controlled Clinical Trial"[Publication Type]) OR "Randomized Controlled Trials as Topic"[Mesh]) OR "Random Allocation"[Mesh]) OR "Double-Blind Method"[Mesh]) OR "Single-Blind Method"[Mesh]) OR "Clinical Trial"[Publication Type]) OR ("clinical trial"[Publication Type] OR "clinical trials as topic"[MeSH Terms] OR "clinical trials"[All Fields])) OR "Placebos"[Mesh]) OR ("placebos"[MeSH Terms] OR "placebos"[All Fields]	2044316

	OR "placebo"[All Fields])) OR ("random allocation"[MeSH Terms] OR ("random"[All Fields] AND "allocation"[All Fields]) OR "random allocation"[All Fields] OR "random"[All Fields])	
**#4**	**#1** AND **#2** AND #3	24

WEB OF SCIENCE

Number of localized studies: 39
Limits: -
Number of studies after applying limits: 39

	**Descriptors**	Number of studies reached
**#1**	TS=("Polycystic Ovary Syndrome") OR TS=("Ovary Syndrome, Polycystic") OR TS=("Syndrome, Polycystic Ovary") OR TS=("Polycystic ovary disease") OR TS=("Stein-Leventhal Syndrome") OR TS=("Stein Leventhal Syndrome") AND TS=("Syndrome, Stein-Leventhal") OR TS=("Sclerocystic Ovarian Degeneration") OR TS=("Ovarian Degeneration, Sclerocystic") OR TS=("Sclerocystic Ovary Syndrome") OR TS=("Polycystic Ovarian Syndrome") AND TS=("Ovarian Syndrome, Polycystic") OR TS=("Polycystic Ovary Syndrome 1") OR TS=("Sclerocystic Ovaries") OR TS=("Ovary, Sclerocystic") OR TS=("Sclerocystic Ovary") OR TS=("hyperandrogenism") OR TS=("Hypertrichosis") OR TS=("Hirsutism") OR TS=("PCOS") OR TS=("PCO")	47675
**#2**	TS=("Magnesium") OR TS=("Magnesium sulfate") OR TS=("Magnesium supplementation")	226114
**#3**	TS=(Randomized controlled trial) OR TS=(controlled clinical trial) OR TS=(randomized controlled trials) OR TS=(random allocation) OR TS=(double blind method) OR TS=(single blind method) OR TS=(clinical trial) OR TS=(clinical trials) OR TS=(placebos) OR TS=(placebo) OR TS=(random)	2342578
**#4**	**#1** AND **#2** AND **#3**	39

SCOPUS

Number of localized studies: 101
Limits: -
Number of studies after applying limits: 101

	**Descriptors**	Number of studies reached
**#1**	(TITLE-ABS-KEY (hypertrichosis)) OR (TITLE-ABS-KEY (hirsutism)) OR (TITLE-ABS-KEY (pcos)) OR (TITLE-ABS-KEY (pco)) OR ((TITLE-ABS-KEY (polycystic AND ovary AND syndrome)) OR (TITLE-ABS-KEY (ovary AND syndrome, AND polycystic)) OR (TITLE-ABS-KEY (syndrome, AND polycystic AND ovary)) OR (TITLE-ABS-KEY (polycystic AND ovary AND disease)) OR (TITLE-ABS-KEY (stein-leventhal AND syndrome)) OR (TITLE-ABS-KEY (stein AND leventhal AND syndrome))) OR ((TITLE-ABS-KEY (syndrome, AND stein-leventhal)) OR (TITLE-ABS-KEY (sclerocystic AND ovarian AND degeneration)) OR (TITLE-ABS-KEY (ovarian AND degeneration, AND sclerocystic)) OR (TITLE-ABS-KEY (sclerocystic AND ovary AND syndrome)) OR (TITLE-ABS-KEY (polycystic AND ovarian AND syndrome)) OR (TITLE-ABS-KEY (ovarian AND syndrome, AND polycystic))) OR ((TITLE-ABS-KEY (polycystic AND ovary AND syndrome 1)) OR (TITLE-ABS-KEY (sclerocystic AND ovaries)) OR (TITLE-ABS-KEY (ovary, AND sclerocystic)) OR (TITLE-ABS-KEY (sclerocystic AND ovary)) OR (TITLE-ABS-KEY (hyperandrogenism)) OR (TITLE-ABS-KEY (hypertrichosis)))	66248
**#2**	(TITLE-ABS-KEY (magnesium)) OR (TITLE-ABS-KEY (magnesium AND sulfate)) OR (TITLE-ABS-KEY (magnesium AND supplementation))	429161
**#3**	(TITLE-ABS-KEY (randomized AND controlled AND trial) OR TITLE-ABS-KEY (controlled AND clinical AND trial) OR TITLE-ABS-KEY (randomized AND controlled AND trials) OR TITLE-ABS-KEY (random AND allocation) OR TITLE-ABS-KEY (double AND blind AND method) OR TITLE-ABS-KEY (single AND blind AND method) OR TITLE-ABS-KEY (clinical AND trial) OR TITLE-ABS-KEY (clinical AND trials) OR TITLE-ABS-KEY (placebos) OR TITLE-ABS-KEY (placebo) OR TITLE-ABS-KEY (random))	3958364
**#4**	**#1** AND **#2** AND **#3**	101

COCHRANE

Number of localized studies: 12
Limits: trials
Number of studies after applying limits: 12

	**Descriptors**	Number of studies reached
**#1**	Me ("Polycystic Ovary Syndrome"):ti,ab,kw or ("Ovary Syndrome, Polycystic"):ti,ab,kw or ("Syndrome, Polycystic Ovary"):ti,ab,kw or ("Polycystic ovary disease"):ti,ab,kw or ("Stein-Leventhal Syndrome"):ti,ab,kw or ("Stein Leventhal Syndrome"):ti,ab,kw or ("Syndrome, Stein-Leventhal"):ti,ab,kw or ("Sclerocystic Ovarian Degeneration"):ti,ab,kw or ("Ovarian Degeneration, Sclerocystic"):ti,ab,kw or ("Sclerocystic Ovary Syndrome"):ti,ab,kw or ("Polycystic Ovarian Syndrome "):ti,ab,kw or ("Ovarian Syndrome, Polycystic"):ti,ab,kw or ("Polycystic Ovary Syndrome 1"):ti,ab,kw or ("Sclerocystic Ovaries"):ti,ab,kw or ("Ovary, Sclerocystic"):ti,ab,kw or ("Sclerocystic Ovary"):ti,ab,kw or ("hyperandrogenism"):ti,ab,kw or ("Hypertrichosis"):ti,ab,kw or ("Hirsutism"):ti,ab,kw or ("PCOS"):ti,ab,kw or ("PCO"):ti,ab,kw	5947
**#2**	Me ("Magnesium") or ("Magnesium sulfate"):ti,ab,kw or ("Magnesium supplementation"):ti,ab,kw	42
**#3**	**#1** AND **#2**	12
